# Home-based intensive treatment of chronic radiation-associated dysphagia in head and neck cancer survivors (HIT-CRAD trial)

**DOI:** 10.1186/s13063-022-06832-6

**Published:** 2022-10-22

**Authors:** Hanne Massonet, Ann Goeleven, Leen Van den Steen, Alice Vergauwen, Margot Baudelet, Gilles Van Haesendonck, Olivier Vanderveken, Heleen Bollen, Lisette van der Molen, Fréderic Duprez, Peter Tomassen, Sandra Nuyts, Gwen Van Nuffelen

**Affiliations:** 1 Faculty of Medicine, Department of Neurosciences, Research Group Experimental Oto-Rhino-Laryngology - Delgutology, KU Leuven, Leuven, Belgium; 2 Faculty of Medicine and Health Sciences, University of Antwerp, Antwerp, Belgium; 3 Department of Head and Neck Surgery, Swallowing Clinic, University Hospitals Leuven, Leuven, Belgium; 4 Department of ENT, Swallowing Clinic, University Hospitals Leuven, Leuven, Belgium; 5 Department of Otolaryngology and Head and Neck Surgery, Rehabilitation Centre for Communication Disorders, Antwerp University Hospital, Antwerp, Belgium; 6 Department of Radiation Oncology, Ghent University Hospital, Ghent, Belgium; 7 Faculty of Medicine and Health Sciences, Department of Human Structure and Repair, Ghent University, Ghent, Belgium; 8 Department of Oncology, University Hospitals Leuven, Leuven, Belgium; 9 Department of Head and Neck Oncology and Surgery, The Netherlands Cancer Institute, Amsterdam, The Netherlands; 10 Faculty of Humanities, University of Amsterdam, Amsterdam, The Netherlands; 11 Department of Head and Neck Surgery, Ghent University Hospital, Ghent, Belgium; 12 Faculty of Medicine and Health Sciences, Department of Rehabilitation Sciences, Ghent University, Ghent, Belgium

**Keywords:** Dysphagia, Head and neck cancer, Chemoradiotherapy, Swallowing exercises, Tongue strengthening exercises, Expiratory Muscle Strength Training (EMST), Chin Tuck against Resistance CTAR), McNeill Dysphagia Therapy Program (MDTP), High-definition transcranial direct current stimulation (HD-tDCS), Non-invasive brain stimulation

## Abstract

**Background:**

Chronic radiation-associated dysphagia (C-RAD) is considered to be one of the most severe functional impairments in head and neck cancer survivors treated with radiation (RT) or chemoradiation (CRT). Given the major impact of these late toxicities on patients’ health and quality of life, there is a strong need for evidence-based dysphagia management. Although studies report the benefit of strengthening exercises, transference of changes in muscle strength to changes in swallowing function often remains limited. Therefore, combining isolated strengthening exercises with functional training in patients with C-RAD may lead to greater functional gains.

**Methods:**

This 3-arm multicenter randomized trial aims to compare the efficacy and possible detraining effects of mere strengthening exercises (group 1) with a combination of strengthening exercises and functional swallowing therapy (group 2) and non-invasive brain stimulation added to that combination (group 3) in 105 patients with C-RAD. Patients will be evaluated before and during therapy and 4 weeks after the last therapy session by means of swallowing-related and strength measures and quality of life questionnaires.

**Discussion:**

Overall, this innovative RCT is expected to provide new insights into the rehabilitation of C-RAD to optimize post-treatment swallowing function.

**Trial registration:**

International Standard Randomized Controlled Trials Number (ISRCTN) registry ID ISRCTN57028065. Registration was accepted on 15 July 2021.

## Administrative information

Note: the numbers in curly brackets in this protocol refer to SPIRIT checklist item numbers. The order of the items has been modified to group similar items (see http://www.equator-network.org/reporting-guidelines/spirit-2013-statement-defining-standard-protocol-items-for-clinical-trials/)

**Table:** 

Title {1}	Home-Based Intensive Treatment of Chronic Radiation Associated Dysphagia in head and neck cancer survivors (HIT-CRAD trial)
Trial registration {2a and 2b}.	ISRCTN, ID ISRCTN57028065. Registration was done prospectively and accepted on 15 July 2021.
Protocol version {3}	V.04 15.09.2021
Funding {4}	This study received financial support from Kom Op Tegen Kanker, a non-profit organization. The Swallowing Exercise Aid (SEA) devices were sponsored by Atos Medical. Nestlé decided to sponsor the products for the instrumental and clinical swallowing evaluations.
Author details {5a}	^1^ KU Leuven, Faculty of Medicine, Department of Neurosciences, Research Group Experimental Oto-rhino-laryngology, Leuven, Belgium ^2^ University of Antwerp, Faculty of Medicine and Health Sciences, Antwerp, Belgium ^3^ University Hospitals Leuven, Department of head and neck surgery, Swallowing Clinic, Leuven, Belgium ^4^ University Hospitals Leuven, Department of ENT, Swallowing Clinic, Leuven, Belgium. ^5^ Antwerp University Hospital, Department of Otolaryngology and Head and Neck Surgery, Rehabilitation Centre for Communication Disorders, Antwerp, Belgium ^6^ Ghent University Hospital, Department of Radiation Oncology, Ghent, Belgium 7Ghent University, Faculty of Medicine and Health Sciences, Department of Human Structure and Repair, Ghent, Belgium ^8^ University Hospitals Leuven, Department of Oncology, Leuven, Belgium ^9^ The Netherlands Cancer Institute, Department of Head and Neck Oncology and Surgery, Amsterdam, The Netherlands ^10^ University of Amsterdam, Faculty of Humanities, Amsterdam, The Netherlands ^11^ Ghent University Hospital, Department of Head and Neck Surgery, Ghent, Belgium ^12^ Ghent University, Faculty of Medicine and Health Sciences, Department of Rehabilitation Sciences, Ghent, Belgium
Name and contact information for the trial sponsor {5b}	Antwerp University Hospital.
Role of sponsor {5c}	The funders are not involved in the design of the study and will not have any role during its conduct, analyses interpretation of data or submission of findings.
Trial organization {5d}	A general steering committee is appointed, consisting of the principal investigators of each center. During the term of the study, the Steering Committee shall meet at least twice a year to discuss the conduct of the study. The steering committee has no authority to amend this agreement. No subcommittees are assigned.

## Introduction

### Background and rationale {6a}

Dysphagia is considered to be one of the most severe functional impairments in head and neck cancer (HNC) survivors treated with radiation (RT) or chemoradiation (CRT) [1–3]. Radiation-associated dysphagia (RAD) may occur temporarily as an acute side effect during or immediately after treatment. However, it may also become chronic (C-RAD) or develop several years post-treatment (Late RAD) [4–6].

Reported prevalence rates for RAD extend to 95% during treatment and around 70% 12 months to more than 10 years post-treatment [1, 7]. Acute toxicities such as oedema, mucositis, and xerostomia typically develop during or immediately after CRT, but generally improve in the following months post-treatment [5, 6]. In contrast, tissue fibrosis and loss of muscle fibers (atrophy) may occur and persist long after completion of the treatment due to decreased blood supply to the muscles which can result in a reduced range of motion and strength [8–10]. Disturbed neurotransmission and lower cranial neuropathies are also important late side effects of chemoradiation causing C-RAD [8, 11–13]. These late effects lead to impairment of crucial swallowing structures affecting both the oral and pharyngeal phase of swallowing resulting in malnutrition, dehydration, and aspiration. Further disuse will enhance the deterioration of muscle fibers, causing a vicious cycle affecting the patient’s health and quality of life [1, 2, 14, 15]. Furthermore, the risk for developing life-threatening aspiration pneumonia is high since the majority of dysphagic HNC survivors seems to aspirate silently [1].

New organ-sparing radiotherapy protocols with an increased focus on swallowing muscle dose-volume parameters and prophylactic swallowing exercises may reduce long-term swallow-related toxicities, but prevention stays challenging [16–20]. Given the major impact of these late toxicities on patients’ health and quality of life, there is a strong need for evidence-based dysphagia management [21]. Currently, evidence-based research addressing patient-supported therapy methods is rather scarce and heterogeneous with low levels of evidence, which delays clinical implementation. Previous studies have shown the efficacy of exercises aiming to improve strength of the main muscles involved in swallowing. However, the majority of evidence is derived from neurological patient populations in which tissue fibrosis is not an issue [22, 23]. Moreover, the degree of evidence is low as most studies are non-controlled case series [24, 25]. Therefore, conducting a randomized controlled trial to investigate the efficacy of these strengthening exercises would be a major step forward.

Although studies report the benefit of strengthening exercises [26–28], transference of changes in muscle strength to changes in swallowing function often remains limited [24, 25, 29]. Simply training strength of the swallowing musculature may not necessarily be sufficient to stimulate key central and peripheral adaptations to improve the swallowing function. However, in patients with significant weakness, it may be beneficial to use strength training as a precursor to functional training to build a foundation of force-producing capacity and prepare the neuromuscular system for task-specific activity [30]. Case studies investigating the effect of functional swallowing programs reveal promising results in patients with chronic dysphagia, including C-RAD [31]. Consequently, combining these isolated strengthening exercises with functional training in patients with C-RAD may lead to greater functional gains [30, 32].

In recent years, the role of cortical plasticity in the rehabilitation of the human swallowing motor cortex has become more apparent [33]. In particular, the use of transcranial direct current stimulation (tDCS) has been widely investigated regarding its clinical efficacy, safety, ease of use, and high tolerability among patients [34]. It has been demonstrated that pairing tDCS or high-definition transcranial direct current stimulation (HD-tDCS) with an active motor task enhances the excitability of the targeted motor network. Hence, pairing HD-tDCS with swallowing therapy could stimulate the impaired motor network even more. Recent research in patients with dysphagia indeed shows that tDCS may improve the effectiveness of both isolated strength and functional training [35, 36]. However, its effect in patients with C-RAD has not been investigated yet.

### Objectives {7}

This 3-arm multicenter randomized trial will investigate the effect of state-of-the-art and innovative rehabilitation methods in patients with C-RAD comparing the efficacy and possible detraining effects of mere strengthening exercises (group 1) with a combination of strengthening exercises and functional swallowing therapy (group 2) and non-invasive brain stimulation added to that combination (group 3). The efficacy of the three treatment programs will be determined based on changes in swallowing function, quality of life, and muscle strength. For future clinical implementation, therapy programs must not only be effective but also feasible, tolerable, and sufficiently attractive to keep the patient motivated. Therefore confounding factors and adherence-specific measures will be investigated. The ultimate goal of this randomized trial is clinical implementation of effective therapy programs for C-RAD.

### Trial design {8}

**Fig. 1:**
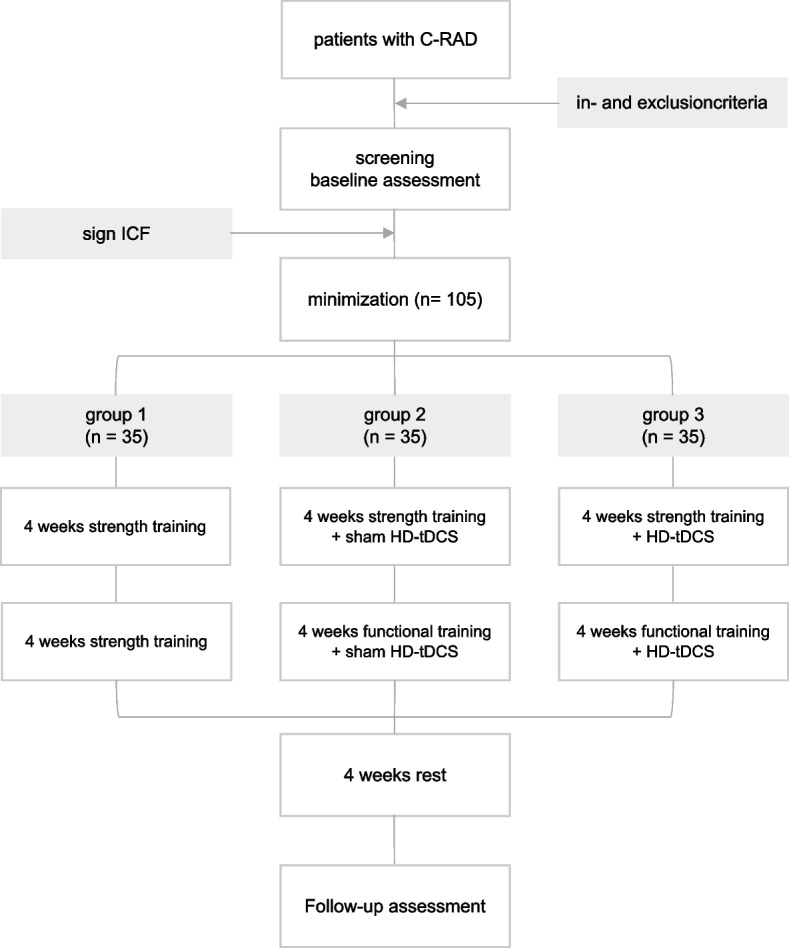
Trial flowchart

This study is a multicenter, parallel design, three-arm, superiority, randomized controlled trial (RCT). An outline of this RCT is presented in Fig. 1. The treatment and outcome measures are presented in Table 1. Measurements will be carried out before treatment, after 4 and 8 weeks of treatment and 4 weeks after the last therapy session. The maximal interval between baseline evaluation and the start of therapy is 2 weeks.

**Table 1:**
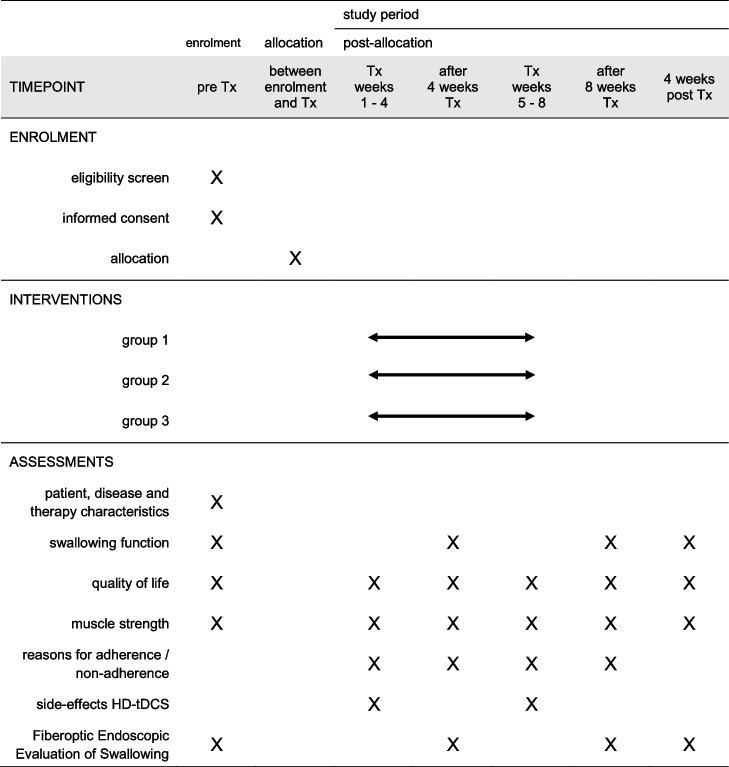
Study visits and assessments

*Tx* treatment

## Methods: participants, interventions and outcomes

### Study setting {9}

In total 105 subjects will be allocated randomly into three groups. This multicenter study will be conducted at the following sites: University Hospital Leuven, Antwerp University Hospital, Sint-Augustinus Hospital and Ghent University Hospital, all located in Belgium.

### Eligibility criteria {10}

#### Inclusion criteria

This study will be carried out in patients with a history of head and neck cancer treated with radiotherapy or chemoradiotherapy meeting the following inclusion criteria:

(A) Diagnosis of C-RAD, present for at least 3 months, based on the Eating Assessment Tool-10 (EAT-10) [37] and/or Functional Oral Intake Scale (FOIS, max level 6 out of 7) [38]
(B) Eligible tumor sites: oral cavity, oropharynx, larynx, hypopharynx and nasopharynx
(C) At least 6 months post oncological treatment

#### Exclusion criteria

Patients with any of the following conditions will be excluded:

(A) History of major surgery within the head and neck region
(B) Recurrent carcinoma in the head and neck region
(C) Neurological history that might adversely affect cognition, muscle strength in the head and neck region, or swallowing function
(D) Dysphagia prior to CRT
(E) Intensive swallowing therapy (> once per week) in the last 6 months
(F) Complete dependency on tube feeding during more than 1 year
(G) Severe frailty following the Clinical Frailty Scale (CFS) (≥ 7) [39]
(H) Malnourished following the Mini Nutritional Assessment (MNA) (< 17) [40]
(I) Related to HD-tDCS: presence of implanted metal or electronic medical devices in the brain or other sites in the body (e.g., Deep Brain Stimulator, Cochlear Implant, pacemaker), history of migraine, epilepsy, brain damage (stroke), or head trauma followed by impairment of consciousness, skin problems (e.g., dermatitis, psoriasis), and use of medication that interferes with non-invasive brain stimulation.

### Who will take informed consent? {26a}

Prior to participation, written informed consent will be obtained from eligible subjects following the most recent Declaration of Helsinki. The possibility of participating in the study will be informed in writing and orally by the researchers of the project.

### Additional consent provisions for collection and use of participant data and biological specimens {26b}

Not applicable. Participant data and biological specimens will not be used in ancillary studies.

## Interventions

### Explanation for the choice of comparators {6b}

To investigate the additional effect of HD-tDCS on changes in swallowing function, muscle strength, and QoL, sham stimulation is applied to participants in the second group. Therefore, the second group is serving as a control group for the application of HD-tDCS.

### Intervention description {11a}

The duration, frequency, and location of the treatment programs are the same in each group. All subjects will practice 4 times a week for 8 weeks. The therapy will take place at home under supervision of a qualified speech-language pathologist (SLP). The exercise program differs according to the group.

The *first group* receives 8 weeks of exercises aiming to improve strength of the swallowing musculature; namely, muscles involved in tongue strength, pharyngeal contraction, and laryngeal elevation/upper esophageal sphincter opening. These strengthening exercises include tongue strengthening exercises (TSE), Chin Tuck Against Resistance (CTAR) in combination with effortful swallowing, and Expiratory Muscle Strength Training (EMST).

**Fig. 2:**
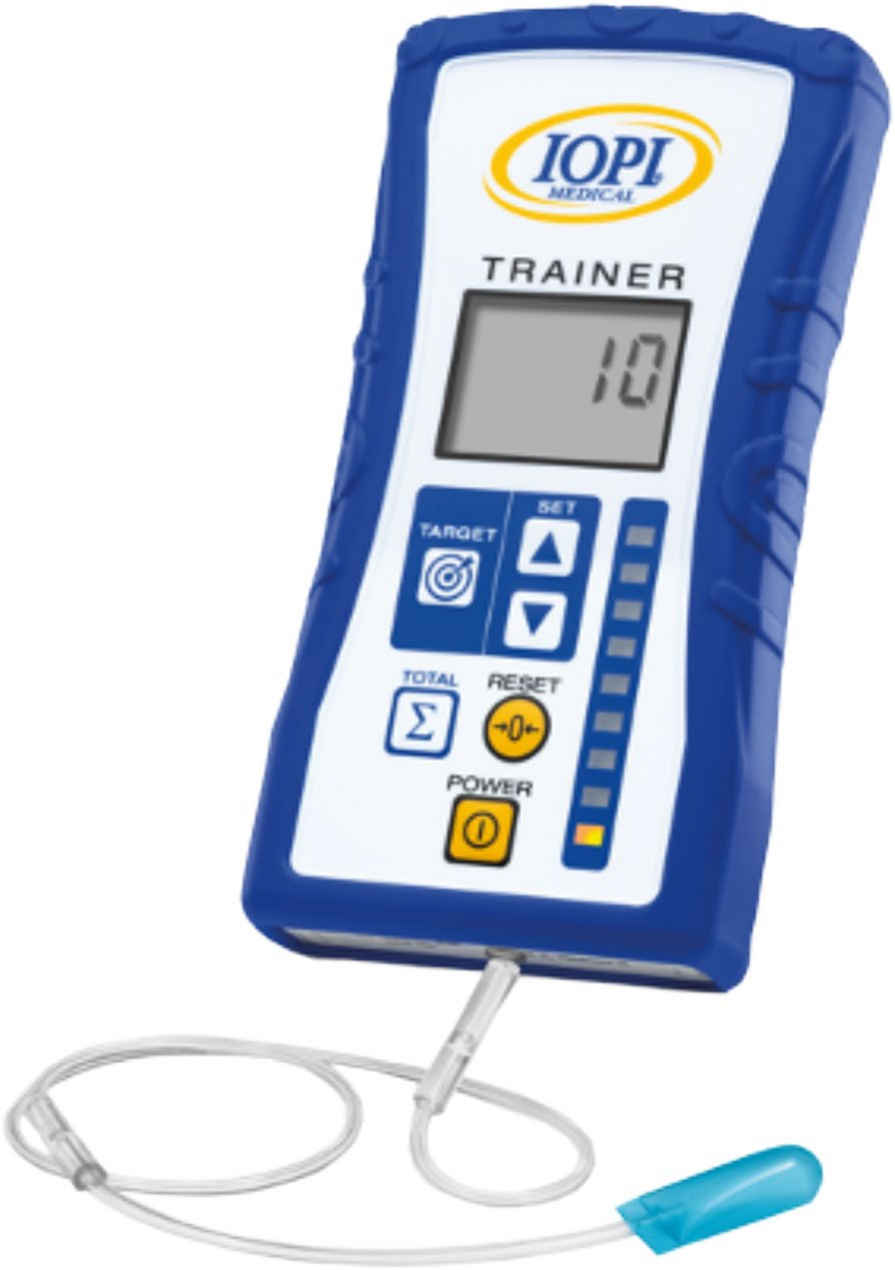
Iowa Oral Performance Instrument, model 3.2. (IOPI Medical, LLC, Woodinville, WA, USA)

**Fig. 3:**
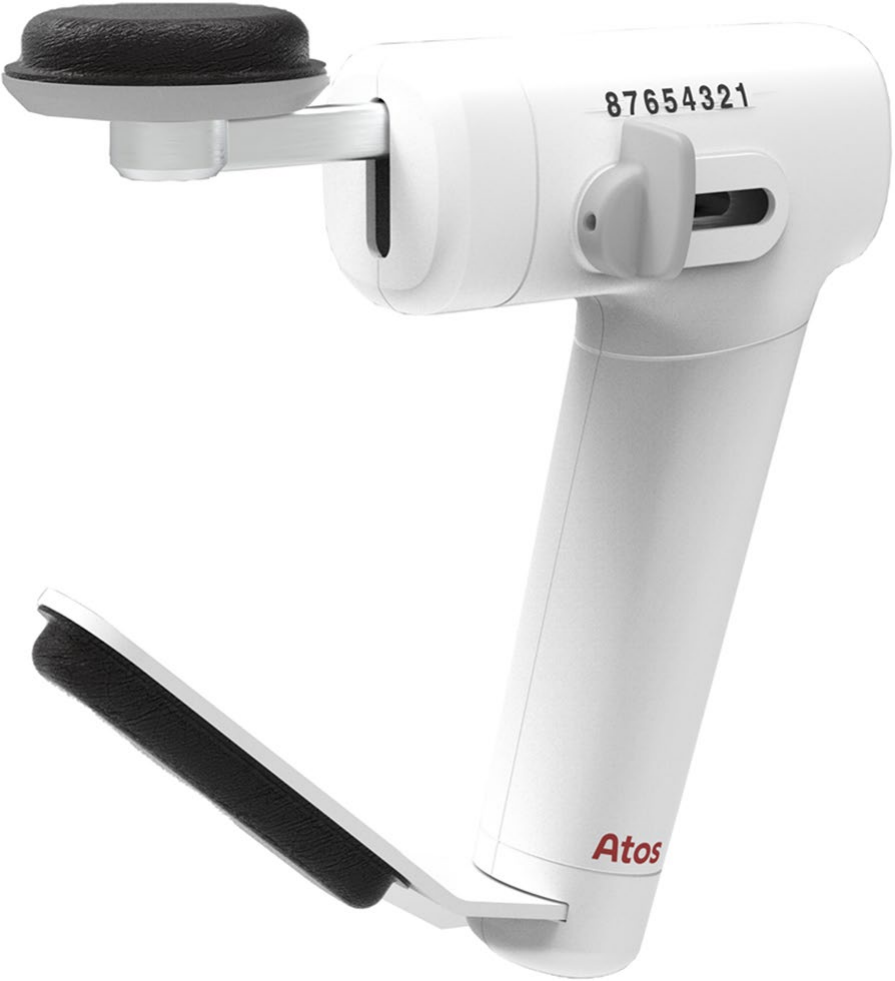
Swallowing Exercise Aid (SEA, courtesy of Atos Medical, www.atosmedical.com). NB: the device is currently only available for research purposes

**Fig. 4:**
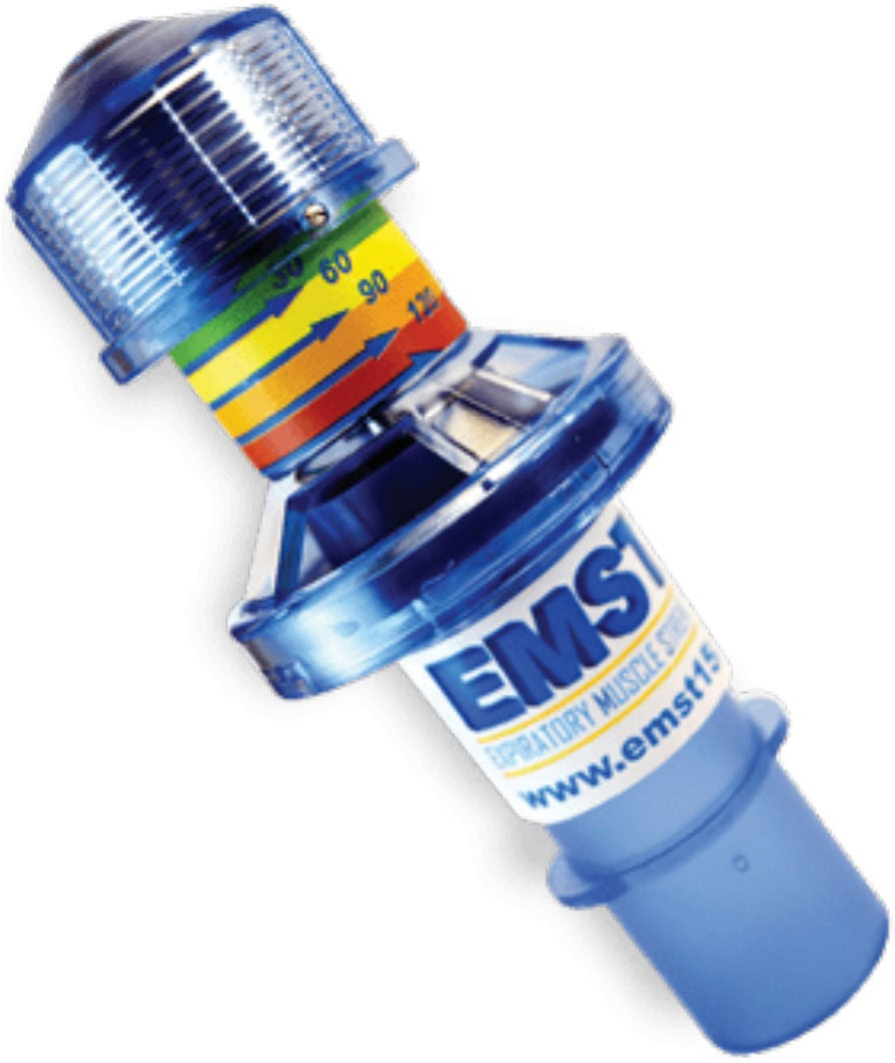
Expiratory Muscle Strength Trainer (EMST150 device, Aspire Products, Gainesville, Florida, USA)

1. First, TSE are performed since tongue strength plays an important role in bolus formation, food mastication, and bolus propulsion. Decreased tongue strength can lead to oral and pharyngeal residue and aspiration [29]. Hence, sufficient tongue strength is a key factor for safe swallowing [23]. The TSE are conducted using the Iowa Oral Performance Instrument™ trainer (model 3.2.) (Fig. 2). The researcher sets the level of resistance (80% of 1 repetition maximum (RM), i.e., the maximum amount of pressure that can be generated in one repetition) which will be adjusted every week. Visual feedback is provided by a series of vertically positioned lights with the top light turning green when the target level is reached. Patients are instructed to place the proximal end of the bulb anteriorly behind the upper teeth at the midline of the palate. They are then asked to squeeze the bulb with the tongue to the palate until the top light turns green and hold this effort for 3 s. Tongue strengthening exercises consist of 120 tongue-palate presses and are divided into 12 sets of 10 repetitions with a 30-s rest between sets.
2. Second, the CTAR exercises are used to strengthen the suprahyoid muscles to improve hyolaryngeal elevation and upper esophageal sphincter opening [41, 42]. These exercises are conducted with the Swallowing Exercise Aid (Fig. 3). This device was developed at the University of Amsterdam by adapting the well-known Therabite™ Jaw Mobilization device. The device has now been further developed and CE marked by ATOS Medical for potential future commercial use and Atos Medical has agreed to make the devices available for the clinical study. It is a handheld device with a chest bar resting on the chest/sternum and a chin bar covered by a chin pad that is placed under the chin which is pressed down on. The dial on the device can be placed in various marked positions that systematically increase resistance ranging from 16.5 N (position 1) to 160 N (position 8). The target level is set weekly at 75% of individualized 1 RM. For each trial, participants are asked to hold the device, place the chest bar on their chest and their chin on the chin pad. They are then instructed to push the chin bar down with their mouths closed and hold it for 3 s. Each CTAR session consists of 150 repetitions divided into 30 sets of five repetitions. Every fifth repetition subjects are asked to push the chin bar towards the chest bar and swallow as hard as they can to practice an effortful swallow.
3. Finally, EMST is performed using the EMST150™ device (Aspire Products, Gainesville, Florida, USA) (Fig. 4), a handheld calibrated, one-way, spring-loaded valve trainer. It has been shown that expiratory muscle strength training improves airway protection by increasing expiratory pressure, resulting in a more efficient cough, and/or improving airway protection by training strength of the suprahyoid muscles [22, 25, 43]. Prior to training, maximal expiratory pressures (MEP) are measured using a spirometer. The training load is set weekly at 75% of MEP. During training, participants sit in a comfortable position and are instructed to take a deep breath, seal their lips around the mouthpiece, hold their cheeks lightly, and exhale as fast and as hard as possible to break the seal. A single-day training session consists of 25 targeted exhalations, performed in 5 sets of 5 repetitions with a 30-s rest between sets.

The different exercises described above alternate during the sessions. Participants will start with TSE the first day and perform CTAR and EMST the following day.

The *second* and *third* group receive a combination of 4 weeks strength training followed by 4 weeks functional swallowing therapy. The strengthening exercises in groups 2 and 3 are the same as in group 1. Functional swallow therapy involves exercise-based practice with swallowing consistencies that challenge the patients’ entire swallowing system by progressively increasing the volume and viscosity of the bolus and hence increasing the required effort to swallow the bolus as described in the McNeil Dysphagia Therapy Program (MDTP) [31]. In MDTP, a food hierarchy is used to gradually increase the volume, consistency, intensity, and timing of swallowing in a standardized manner. Participants start at a certain level and move forward with a series of successful swallows or backwards with a series of unsuccessful swallows. The starting level is considered to be the most advanced level of consistency a patient can swallow safely, determined based on an instrumental swallowing assessment (fiberoptic endoscopic evaluation of swallowing, FEES).

**Fig. 5:**
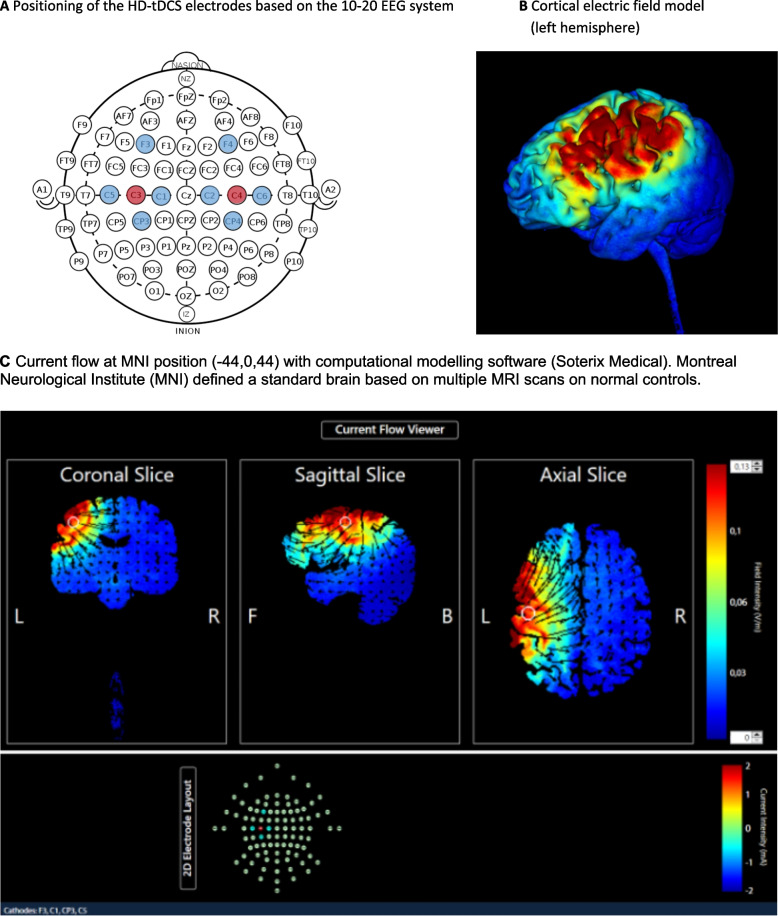
4 × 1 Concentric HD-tDCS. Three return electrodes (blue) are placed at a ring-center-to-ring-center distance of 3 cm from the active electrode (red) and one will be placed further apart to elicit an adequate electric field strength in the mid-inferior lateral section of the precentral gyrus as suggested by computational modelling software (Soterix Medical, New York, NY, United States). The anode will be placed on the swallowing motor cortex, C3 on the left and C4 on the right hemisphere based on the international 10–20 EEG system

High-definition transcranial direct current stimulation (HD-tDCS) (Soterix 1x1 tDCS stimulator Model 1300A, Soterix 4x1-C3A HD-tDCS multichannel device and HD-caps (Soterix, Medical Inc)) will be used in the *third* group to modulate the cortical excitability during training. HD-tDCS is a form of non-invasive brain stimulation using a low positive current applied to the skull by one center anode and 4 return electrodes (4×1 concentric HD-tDCS). Three return electrodes are placed at a ring-center-to-ring-center distance of 3 cm from the active electrode and one will be placed further apart to elicit an adequate electric field strength in the mid-inferior lateral section of the precentral gyrus as suggested by computational modelling software (Soterix Medical, New York, NY, USA) (Fig. 5a, b and c). The center electrode will be placed on the swallowing motor cortex (Brodmann area 4, the mid-inferior lateral section of the primary motor cortex [42]), C3 on the left and C4 on the right hemisphere based on the international 10-20 electroencephalogram (EEG) electrode system (Fig. 5a). The surrounding electrodes will be placed on C1, CP3, C5, and F3 on the left hemisphere and C2, CP4, C6, and F4 on the right hemisphere. The stimulation will be administered bilaterally but not simultaneously. Each hemisphere will be stimulated alternately for 2 consecutive days. A constant direct current of 2 mA is applied for 20 min each session with a fade-in/fade-out of 1 min. In the sham group (group 2), the same protocol will be applied; however, the 2 mA current will be ramped up for only 1 min and then switched off, which will produce an initial tingling sensation but no significant changes in cortical excitability [44]. HD-tDCS treatment is provided in a total of 32 sessions and all sessions are completed within 8 weeks (4 sessions per week). Patients are randomized into a real treatment HD-tDCS group (group 3) or a sham (placebo) group (group 2) and are blinded to the group in which they are in. Necessary safety measures and internationally recommended exclusion criteria will be taken into account [44–48].

### Criteria for discontinuing or modifying allocated interventions {11b}

tDCS has been thoroughly investigated and is considered to be safe [46]. The tolerability of HD-tDCS is supported by studies using intensities as high as 2.0 mA for up to 20 min [49]. The possible side effects of HD-tDCS are assessed systematically in this study using a standardized questionnaire. Expected side effects are mild to moderate and limited to skin lesions under the electrodes, mild tingling or itching sensations, and transient redness. If the participant experiences a high degree of pain or discomfort, a rest day will be scheduled. Patients will still perform strength or functional training exercises that day but without receiving HD-tDCS. When the described complaints remain present, the treatment will be stopped after further consideration with the patient and members of the study group. All reported side effects related to the HD-tDCS will be analyzed and reported in future publications.

### Strategies to improve adherence to interventions {11c}

Each therapy session will be supervised by a certified SLP to maximize participants’ adherence and to guarantee correct performance of the exercises and the therapy protocol. To limit the additional burden for the participants, therapy sessions will preferentially take place at the participant’s home.

### Relevant concomitant care permitted or prohibited during the trial {11d}

During the study period, other forms of dysphagia rehabilitation are not allowed. There are no other restrictions regarding concomitant care or interventions.

### Provisions for post-trial care {30}

There is no anticipated harm associated with or compensation for trial participation.

### Outcomes {12}

The outcome measures in this RCT are divided into four main domains: (1) Swallowing function, (2) Quality of life, (3) Muscle strength, and (4) Adherence/non-adherence.

#### Swallowing function

The primary outcome measure of this study is a change in functional oral intake based on scores from the FOIS [38], measured after 8 weeks of training. The FOIS is considered a reliable and valid 7-point severity scale completed by a health care professional. The scale ranges from no oral intake (1) to total oral intake with no restrictions (7) [38]. Secondary outcome measurements for swallowing function include the Mann Assessment of Swallowing Ability-Cancer (MASA-C), a reliable and valid scale which is sensitive to differences in swallowing performance in HNC patients with or without dysphagia [50]. A thorough FEES examination is carried out at baseline, after 4 and 8 weeks of training and 4 weeks after the last training session. The EAT-10, a self-administered outcome instrument for dysphagia is questioned [37], as well as visual analog scales (VAS) examining the swallowing difficulties and fear experienced by the patient. For the latter, 100-mm scales are used. Oral intake is further investigated using the Food Intake Level Scale (FILS), a 10-point scale developed to document the functional level of oral intake of food and liquid and the International Dysphagia Diet Functional Diet Scale (IDDSI—FDS), a new functional outcome scale intended to reflect the severity of oropharyngeal dysphagia, as represented by the recommended IDDSI level for eating and drinking [51, 52]. The Mini Nutritional Assessment (MNA-SF, Nestlé) is used to evaluate the nutritional status [40]. Both the FEES and the MASA-C are conducted with 2 × 5 ml and 1 × 10 ml thin liquid (IDDSI 0), 2 × 5 ml and 1 × 10 ml thickened liquid (IDDSI 2), 2 × 5 ml and 1 × 10 ml semi-solid (IDDSI 3), and 1 solid bolus (IDDSI 7). Outcome measurements for FEES include the Penetration Aspiration Scale (PAS) [53], the Pooling score [54], and the Dynamic Imaging Grade of Swallowing Toxicity (DIGEST) scores [55]. All FEES examinations will be recorded for data analysis. Twenty randomly selected FEES recordings will be assessed blindly by 2 experts in mutual agreement. One expert will assess 20% of these recordings twice to determine intra-rater agreement. All swallowing function outcome measures are examined at baseline, after 4 and 8 weeks of training, and 4 weeks after the last training session.

#### Quality of life

Swallowing-related quality of life is examined using the Dutch version of the Dysphagia Handicap Index (DHI) [56]. This questionnaire is completed at baseline, weekly during training, after 4 and 8 weeks of training, and 4 weeks after the last training session.

#### Muscle strength

*Maximal tongue-palate pressure* is assessed using the IOPI Pro, model 3.1. (IOPI Medical LLC, Woodinville, WA, USA). Maximal isometric pressure (MIP) can be measured at the anterior and posterior position. The anterior position is the same as described previously for the tongue strengthening exercises [28]. In the posterior position, the distal tip of the bulb is placed at the transition between the hard and soft palate, at the midline of the palate. The participants are instructed to push the bulb as hard as possible. The highest value of three trials is considered the MIP.

*Maximal strength of the suprahyoid muscles* (SHM) is measured with a dynamometer (Microfet™, Biometrics, Almere, The Netherlands). Participants are instructed to place their chin on the chin bar and keep their mouths and teeth closed while pressing their chin down as hard as possible. The highest value of three trials (in Newtons, N) is considered the maximal isometric chin-tuck strength. Seventy-five percent of maximal isometric chin-tuck strength will be used to determine the target level for training ranging from 16.5 N (position 1) to 160 N (position 8).

*Maximal expiratory pressure* (MEP) is measured using the RP Check (MD Diagnostics, UK). Participants are instructed to “fill their lungs as much as possible, place their lips around the mouthpiece and blow out as fast and as hard as possible.” The best out of 3 trials is considered the MEP.

The strength measurements are performed at baseline, weekly during training, and 4 weeks after the last training session.

#### Adherence / non-adherence

Reasons for non-adherence will be recorded after each session through standardized questions: “how much difficulty did you have completing the session?”, “What factors made therapy difficult?”, and “any other important message?”. Patients’ experiences and suggestions are investigated by means of weekly interviews with standardized open-ended questions. The possible impact of the emotional relationship between client and therapist as well as the degree of agreement on therapy goals and tasks is assessed after 8 weeks of training using the Dutch version of the work-alliance questionnaire (WAV-12) [57]. Finally, side effects of HD-tDCS are surveyed and documented after each session by means of standardized questions.

Conclusions will be based on the comparison of the change in score at 8 weeks versus baseline in the 3 arms. Averages and standard deviations will be used as aggregation methods for all outcome measurements.

### Confounders

#### Patients, disease and therapy characteristics

Patient and situational characteristics in particular age, gender, educational level, social status, personality traits (using the NEO-Five Factor Inventory (NEO-FFI) [58]), frailty (using the Clinical Frailty Scale (CFS) [39]) handedness [59], and dental condition (using the Oral Health Assessment Tool (OHAT)) are examined at baseline [60]. Disease characteristics include location and stage of the carcinoma, TNM classification, HPV status (based on p16 immunohistochemistry; cut-off > 70% cytoplasmatic and nuclear staining [61]), complaints of xerostomia [62], and thicky saliva (VAS). Treatment-related information is assessed, i.e., chemotherapy, fractionation, bilateral neck irradiation, duration, and time post-treatment. This information is gained at baseline by radiation oncologists, head and neck surgeons, and otolaryngologists.

#### Attitudes about exercises

Participants’ attitudes on exercises are investigated weekly during training using a standardized questionnaire developed by Sluijs et al. [63].

### Participant timeline {13}

All participants will be assessed using the scales described above at baseline, weekly during therapy, after 4 and 8 weeks of therapy, and 4 weeks after the last therapy session. Table 1 gives an overview of study assessments at different timepoints.

### Sample size {14}

The primary conclusions of this study will be based on a linear mixed effects model with post hoc comparisons. For the sample size calculation, this analysis was simplified to 3 pairwise comparisons at week 8 by means of independent samples *t*-tests. Based on Carnaby-Mann et al., we assume a change in Functional Oral Intake Scale (FOIS) after 8 weeks of 2.5 with a standard deviation (SD) of 1.7 in group 2 [31]; based on our own experience, we expect little effect in group 1 (change = 1 with same SD). With these numbers in mind, we want to be able to detect a difference of 1.5 change in FOIS between any 2 groups, with a SD of 1.7. Therefore, we need to include 31 patients per group to achieve 80% power at a significance level of 0.017. A Bonferroni correction for multiple testing was applied to the significance level, as three comparisons will be made (between the 3 groups). To cover for drop-out, we add 4 extra subjects per group. In conclusion, a total sample size of 105 participants (*n* = 35/group), taking into account drop-outs, is needed to demonstrate a statistically significant difference. The software PASS 11 is used to estimate the sample size.

### Recruitment {15}

Recruitment of eligible subjects will be done by the appointed researchers during the multidisciplinary oncological follow-up consultations in the University hospitals of Leuven, Antwerp, and Ghent and in the Sint-Augustinus Hospital. All patients treated with primary RT or CRT who are at least 6 months post-treatment are referred to the researchers for completion of the EAT-10 and FOIS. If a patient appears eligible to participate based on these measurements, the study will be explained in more detail. Participants who are interested in taking part are further assessed for eligibility. Patients who are in doubt about participation receive a folder with information about the study and contact details. If a participant agrees to participate, the informed consent is signed before the baseline measurements are performed by the researcher of each center.

The participating centers have a high level of expertise in the field of head and neck cancer and dysphagia research. Data collection in these hospitals enables the study to include an adequate amount of patients to obtain sufficient power.

## Assignment of interventions: allocation

### Sequence generation {16a}

Patients who are eligible according to the inclusion criteria and signed the informed consent form are randomly assigned to one of the three groups with a 1:1:1 allocation using the program Qminim, an online minimization service supported by the Information and Communication Technology (ICT) Unit of the Antwerp University Hospital. Selected minimization factors include center, FOIS score (level 1 or 2 for more than 3 months, level 1 or 2 for less than 3 months, levels 3 to 6), and time between completion RT or CRT and beginning of the therapy program (< 1 year post Tx, between 1 and 5 years post Tx, > 5 years post Tx).

### Concealment mechanism {16b}

The assignment is performed automatically in real-time based on these criteria. Researchers of each center have access to the program but do not have any precognition of the randomization. Since this is a single-blinded study, minimization and outcome assessment for groups 2 and 3 will be blinded for the participants.

### Implementation {16c}

The SLPs and medical staff who enrol eligible participants will enter the minimization factors in Qminim.

## Assignment of interventions: blinding

### Who will be blinded {17a}

Since this is a single-blinded study, minimization and outcome assessment for groups 2 and 3 will be blinded for the participants. The statistician and 2 independent doctors who will rate the FEES videos after completion of the study are blinded to the real or sham HD-tDCS intervention. No other parties are blinded to the real or sham HD-tDCS intervention.

### Procedure for unblinding if needed {17b}

Not applicable. The allocated intervention will not be revealed to participants during the trial.

## Data collection and management

### Plans for assessment and collection of outcomes {18a}

Before recruitment, all researchers will receive an instruction manual and a presentation of the study to ensure consistency and standardization of data collection and assessment. All data are recorded in case report forms (CRFs) by the data collectors during baseline, treatment, and upon completion of the treatment and follow-up phase. Physicians will be trained to apply the assessment tools required for this study ensuring accurate and thorough collection of data. The CRFs will be read repeatedly before the start of the study. Uniform agreements will also be made in advance regarding the use of the strength training equipment. Furthermore, the SLPs completed a course to obtain certification for treating patients following the MDTP guidelines and attended several training sessions for administering HD-tDCS.

### Plans to promote participant retention and complete follow-up {18b}

Participants receive daily counselling and are motivated to continue their participation. The home-based treatment protocol at a frequency of four times a week is likely to have a positive impact on patients’ adherence. Appointments are scheduled and shared well in advance. Hospital appointments are reminded at least 2 days in advance. Participants are followed up once, 4 weeks after the last therapy session. This follow-up appointment, if possible, is scheduled together with other follow-up appointments in the hospital.

### Data management {19}

Datasets are entered and stored in a non-publicly available repository. All clinical record forms are managed using REDCap (Research Electronic Data Capture), supported by the ICT department of the Antwerp University Hospital [64]. Redcap is a secure web application designed to support data capture for research studies. Both the printed CRFs and electronic data will be locked in order to avoid that adjustments can be made after the data has been recorded. The researchers of each participating institution have access to the data of their patients. The Principal Investigator and the first author have access to all collected data.

### Confidentiality {27}

All data is pseudonymized, encoded, and securely stored for 30 years. Identifying patients’ information will be saved on the secure server of each research center which only the researcher of the particular center will have access to.

### Plans for collection, laboratory evaluation, and storage of biological specimens for genetic or molecular analysis in this trial/future use {33}

This RCT does not require collection of biological specimens.

## Statistical methods

### Statistical methods for primary and secondary outcomes {20a} and methods for additional analyses (e.g. subgroup analyses) {20b}

The primary conclusions of this project will be based on the intent to treat population. All patients will be analyzed in the treatment group they were assigned to. Patients who withdraw consent for use of their data will not be included in any analysis.

Data will be analyzed using a repeated measures analysis with post hoc testing, using the most recent version of IBM SPSS Statistics (V27.0) and R software. Evaluation of the primary endpoint will be performed using a linear mixed effects model with a random intercept per subject and fixed effects of group, time (categorical with 4 categories: baseline, 4 weeks, 8 weeks, and 12 weeks), and interaction between time and group. Based on this model, post hoc comparisons will be performed. The primary conclusion will be based on the comparison of the change at 8 weeks versus baseline in the three arms. Bonferroni-Holm correction for multiple testing will be applied here.

Additionally, linear, logistic, and binary mixed effects models with random intercept, fixed group, time (categorical), and time by group interaction will be used to evaluate the differences in all secondary outcome measures over time in the three groups. For linear mixed effects models coefficients, standard errors and p-values will be reported. For logistic and ordinal logistic mixed models, odds ratios, 95% confidence intervals, and p-values will be given.

Post hoc comparisons will be made based on these models. Confounders can be added to the model.

### Interim analyses {21b}

To avoid introducing bias, no interim analyses will be performed.

### Methods in analysis to handle protocol non-adherence and any statistical methods to handle missing data {20c}

For all endpoints, data is assumed to be missing at random (MAR) and thus will be ignored in the analyses. By using mixed effects models for the analysis, we can incorporate all information on the available time points. If more than 15% of an outcome over all timepoints is missing, a sensitivity analysis will be conducted by using multiple imputation with 20 imputations based on all available information. Results of the original analysis of the available cases will be interpreted in the context of the sensitivity analysis.

Information that is unavailable due to withdrawal of consent will not be considered “missing” and will not be included in the calculation of the percentage of missing data described above.

### Plans to give access to the full protocol, participant-level data, and statistical code {31c}

The dataset, full protocol, and statistical code will not be made publicly available.

## Oversight and monitoring

### Composition of the coordinating center and trial steering committee {5d}

A collaborative agreement was signed between the various participating centers. In this agreement, the roles and responsibilities of the different centers and the researchers per center were clearly formulated and agreed upon.

### Composition of the data monitoring committee, its role and reporting structure {21a}

Poreisz et al. summarized the adverse events of 567 tDCS sessions over motor and non-motor cortical areas, showing relatively minor adverse effects. None of the subjects requested to terminate the stimulation or needed medical assistance during or after the application [65]. Side effects of HD-tDCS described in the literature also include mild tingling, temporary redness, and mild pain due to the electrodes [49]. As the experimental interventions are very low-risk, no data monitoring committee will be formed.

### Adverse event reporting and harms {22}

Participants are encouraged to report any adverse events immediately to the research team. They have the possibility of withdrawing their participation at any time in case serious adverse events occur. Detailed records of all adverse events are maintained and reported in accordance with legal and regulatory requirements. Serious adverse events are reviewed by an appropriate committee for the monitoring of trial safety. Suspected unexpected serious adverse reactions (SUSAR) are identified and fully reported to the regulatory authority and the central ethics committee within the required timelines.

### Frequency and plans for auditing trial conduct {23}

The sponsor and funder are not involved in the design of the study and will not have any role during its conduct, analyses, interpretation of data, or submission of findings. The course of the study will be reported to them annually.

### Plans for communicating important protocol amendments to relevant parties (e.g., trial participants, ethical committees) {25}

All changes are first discussed during the steering committee meetings with the researchers and principal investigators of each center. For every change in the study protocol, an amendment is submitted to the central and local ethical committees.

### Dissemination plans {31a}

Professional caregivers will be informed by peer-reviewed journals and presentations at different national and international conferences. Patients will be informed through patient organizations and publications in patient information magazines. Finally, a conference open for caregivers and patients will be organized to disseminate the findings of the project.

## Discussion

Since RAD is considered to be one of the most severe long-term toxicities, affecting patient’s health and quality of life, there is a strong need for evidence-based dysphagia management. Over the past few years, several studies have indicated that strength training programs using a high number of repetitions and high resistance and frequency do have the potential to improve muscle strength and function in these patients. In addition, an increase in strength seems to result in increased swallowing safety or efficiency. However, it should be noted that significant changes in functional oral intake are not yet observed in the head and neck cancer population. These results indicate that strength training can certainly be a treatment modality in itself, but perhaps may elicit even greater functional gains when used as a precursor to functional swallowing therapy. Therefore, this study will compare the efficacy of mere strength training with a combination of strength and functional training based on the guidelines of the McNeill Dysphagia Therapy Program. Furthermore, by providing HD-tDCS during this combined strength-functional training, we want to explore whether non-invasive brain stimulation could enhance the effect of both isolated strength and functional training in this challenging population. Several studies have investigated the possible application of tDCS to modulate the swallowing motor cortex in neurogenic populations. So far, relatively little attention has been paid to the use of non-invasive brain stimulation in non-neurogenic populations. Therefore, it remains uncertain whether HD-tDCS can also induce changes in the connectivity of the neural network in the head and neck cancer population.

It is expected that by comparing the efficacy of these innovative therapy programs, this multicenter randomized trial will eventually result in clinical guidelines concerning the rehabilitation of C-RAD to address the long-term swallowing-related toxicities resulting from RT/CRT.

## Trial status

Before recruitment, there has been a try-out including 4 patients. The try-out started at the beginning of July 2021 and ran until the end of September 2021. Data collection of the main study started in October 2021 and will be completed January 2025.

## Data Availability

The dataset will be kept by the research team after finishing the study. All Principal Investigators will have access to the collected dataset. The data will not be made publicly available since they will contain patient data. The datasets are available from the corresponding author on reasonable request.

## Abbreviations

C-RAD: Chronic radiation-associated dysphagia (C-RAD)
CFS: Clinical Frailty Scale
CRF: Case report forms
CRT: Chemoradiation
CTAR: Chin Tuck Against Resistance
DHI: Dysphagia Handicap Index
DIGEST: Dynamic Imaging Grade of Swallowing Toxicity
EAT-10: Eating Assessment tool-10
EEG: Electroencephalogram
EMST: Expiratory Muscle Strength Training
FEES: Fiberoptic endoscopic evaluation of swallowing
FILS: Food Intake Level Scale
FOIS: Functional Oral Intake Scale
HD-tDCS: High-definition transcranial direct current stimulation
HNC: Head and neck cancer
HPV: Human papillomavirus
ICT: Information and Communication Technology
IDDSI-FDS: International Dysphagia Diet Functional Diet Scale
IDDSI: International Dysphagia Diet Inventory
IOPI: Iowa Oral Performance Instrument
ISRCTN: International Standard Randomized Controlled Trials Number
MASA-C: Mann Assessment for Swallowing Ability – Cancer
MDTP: McNeill Dysphagia Therapy Program
MEP: Maximal expiratory pressures
MNI: Montreal Neurological Institute
NEO-FFI: NEO-Five Factor Inventory
OHAT: Oral Health Assessment Tool
PAS: Penetration Aspiration Scale
RCT: Randomized controlled trial
Redcap: Research Electronic Data Capture
RM: Repetition maximum
RT: Radiotherapy
SD: Standard deviation
SEA: Swallowing Exercise Aid
SHM: Maximal strength of the suprahyoid muscles
SLP: Speech-language pathologist
SNAQ: Short Nutritional Assessment Questionnaire (SNAQ)
SUSAR: Suspected unexpected serious adverse reactions
tDCS: Transcranial direct current stimulation
TSE: Tongue strengthening exercises
Tx: Treatment
VAS: Visual analog scale
WAV-12: Work-alliance questionnaire
